# Divergent Genomic and Epigenomic Landscapes of Lung Cancer Subtypes Underscore the Selection of Different Oncogenic Pathways during Tumor Development

**DOI:** 10.1371/journal.pone.0037775

**Published:** 2012-05-21

**Authors:** William W. Lockwood, Ian M. Wilson, Bradley P. Coe, Raj Chari, Larissa A. Pikor, Kelsie L. Thu, Luisa M. Solis, Maria I. Nunez, Carmen Behrens, John Yee, John English, Nevin Murray, Ming-Sound Tsao, John D. Minna, Adi F. Gazdar, Ignacio I. Wistuba, Calum E. MacAulay, Stephen Lam, Wan L. Lam

**Affiliations:** 1 Department of Integrative Oncology, British Columbia Cancer Research Centre, Vancouver, British Columbia, Canada; 2 Department of Pathology, The University of Texas MD Anderson Cancer Center, Houston, Texas, United States of America; 3 Departments of Thoracic/Head and Neck Medical Oncology, The University of Texas MD Anderson Cancer Center, Houston, Texas, United States of America; 4 Department of Surgery, Vancouver General Hospital, Vancouver, British Columbia, Canada; 5 Department of Pathology, Vancouver General Hospital, Vancouver, British Columbia, Canada; 6 Department of Medical Oncology, British Columbia Cancer Agency, Vancouver, British Columbia, Canada; 7 Department of Pathology, University Health Network – Princess Margaret Hospital and Ontario Cancer Institute, University of Toronto, Toronto, Ontario, Canada; 8 Hamon Center for Therapeutic Oncology Research, University of Texas Southwestern Medical Center, Dallas, Texas, United States of America; University of Barcelona, Spain

## Abstract

For therapeutic purposes, non-small cell lung cancer (NSCLC) has traditionally been regarded as a single disease. However, recent evidence suggest that the two major subtypes of NSCLC, adenocarcinoma (AC) and squamous cell carcinoma (SqCC) respond differently to both molecular targeted and new generation chemotherapies. Therefore, identifying the molecular differences between these tumor types may impact novel treatment strategy. We performed the first large-scale analysis of 261 primary NSCLC tumors (169 AC and 92 SqCC), integrating genome-wide DNA copy number, methylation and gene expression profiles to identify subtype-specific molecular alterations relevant to new agent design and choice of therapy. Comparison of AC and SqCC genomic and epigenomic landscapes revealed 778 altered genes with corresponding expression changes that are selected during tumor development in a subtype-specific manner. Analysis of >200 additional NSCLCs confirmed that these genes are responsible for driving the differential development and resulting phenotypes of AC and SqCC. Importantly, we identified key oncogenic pathways disrupted in each subtype that likely serve as the basis for their differential tumor biology and clinical outcomes. Downregulation of *HNF4α* target genes was the most common pathway specific to AC, while SqCC demonstrated disruption of numerous histone modifying enzymes as well as the transcription factor *E2F1. In silico* screening of candidate therapeutic compounds using subtype-specific pathway components identified HDAC and PI3K inhibitors as potential treatments tailored to lung SqCC. Together, our findings suggest that AC and SqCC develop through distinct pathogenetic pathways that have significant implication in our approach to the clinical management of NSCLC.

## Introduction

Lung cancer is the leading cause of cancer-related deaths worldwide and despite current treatments, prognosis remains poor, with a five year survival of <18% [1], [2], [3]. Non-small cell lung cancer (NSCLC) and small cell lung cancer (SCLC) are the two main histologic groups. SCLC arises mainly in the central airways while NSCLC may occur centrally or peripherally. The differing pathology of the two types is reflected in their clinical management.

NSCLC is a heterogeneous disease with squamous cell carcinoma (SqCC) and adenocarcinoma (AC) being the predominant histological subtypes. Traditionally, these subtypes have been treated as a single disease entity with treatment strategies determined solely by disease stage. However, recent evidence from clinical trials has demonstrated that histological subtypes of NSCLC respond differently to both targeted drugs and newly developed chemotherapies, possibly related to differences in cell derivation and pathogenetic origins [3], [4], [5], [6], [7], [8], [9]. One of the most striking examples is the folate antimetabolite Pemetrexed, which exhibits superior efficacy and is restricted for use in patients with non-SqCC, presumably due to the higher expression of thymidylate synthase in SqCC tumors [9]. Likewise, numerous studies have associated a higher response rate upon treatment of AC with the EGFR tyrosine kinase inhibitors Gefitinib and Erlotinib, reflecting the higher prevalence of *EGFR* mutations in this subtype [6], [10]. These discrepancies in tumor biology and clinical response highlight the need to determine the underlying genetic, epigenetic and metabolic similarities as well as differences between the NSCLC subtypes in order to define more appropriate avenues for therapeutic intervention.

Initial gene expression profiling studies were able to segregate AC and SqCC tumors into their respective histologic groupings based on multi-gene models; however, critical events in tumorgenesis may be masked by reactive changes when examining expression profiles alone [11], [12], [13]. Conversely, DNA copy number or DNA methylation changes corresponding with gene expression changes are often regarded as evidence of causality. Such DNA level changes are critical deregulation events driving progression and other cancer phenotypes [14], [15], [16]. Since SqCC and AC are thought to develop from distinct cell lineages in different regions of the lung, the range of genetic alterations required for tumor initiation may occur in a lineage-restricted manner. For example, the amplification of the lineage survival oncogenes *SOX2* and *TITF1/NKX2-1* have recently been identified as key events specific to the development of lung SqCC and AC, respectively [17], [18]. However, these genes alone are insufficient to explain the phenotypic diversity of the subtypes, suggesting that the vast majority of genes responsible for their differential development remain unknown. Although genetic and epigenetic differences between SqCC and AC have been described, low genome coverage and/or small sample sizes have been limiting [19], [20], [21], [22], [23], [24].

In this study, we performed the first large-scale analysis of primary NSCLC tumors (261 total –169 AC and 92 SqCC), integrating high resolution DNA copy number, methylation and gene expression profiles to identify critical subtype-specific molecular features. The characterization of the genomic and epigenomic landscapes of AC and SqCC revealed an astounding number of differences at the DNA level with subsequent gene expression changes that are selected for during subtype-specific lung tumor development. Importantly, we identified key oncogenic pathways disrupted by these alterations that likely serve as the basis for differential behaviors in tumor biology and clinical outcomes. Lastly, through prognostic analysis and *in silico* screening of candidate therapeutic compounds using subtype-specific pathway components, we show how these new findings may influence our approach to the clinical management of NSCLC.

## Results

### Assessment of global genomic instability in AC and SqCC

**Figure 1:**
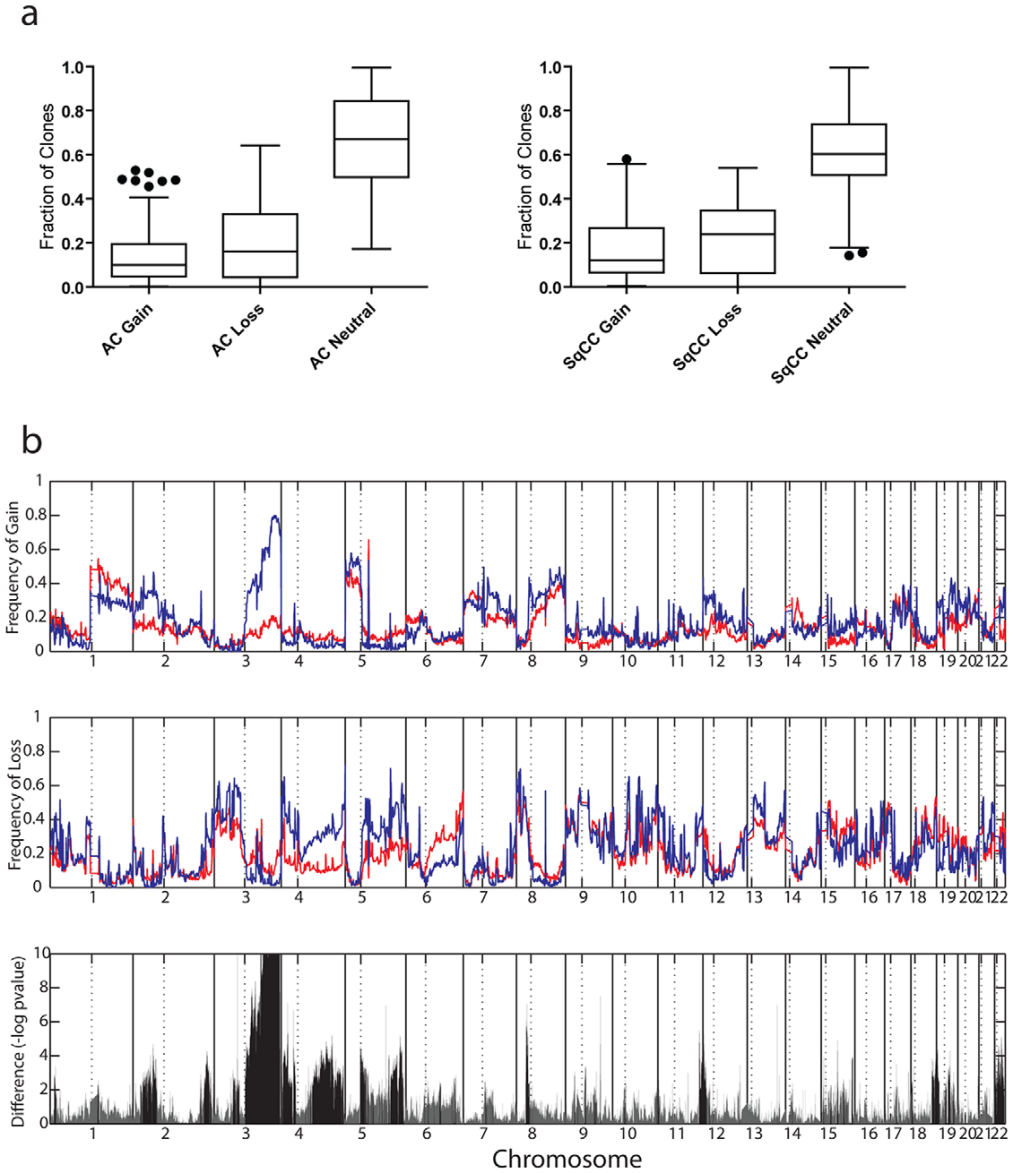
Genomic landscapes of lung AC and SqCC. (a) Percentage of clones of each state in both subtypes. Box plots illustrate the percentage of clones with status −1 (loss/deletion), 0 (neutral), and +1 (gain/amplification) in each of the subtypes. Percentages were calculated for each sample and for each status. These plots demonstrate the similarity in total genome alteration percentages between AC and SqCC tumors and suggest that recurrently altered regions of genome are the result of selection rather than a higher frequency of gain or loss of DNA in either subtype. (b) Alteration frequencies for 169 AC (red) and 92 SqCC (blue) tumors are displayed across the entire human genome. Solid vertical black lines represent chromosome boundaries whereas the dotted black lines represent chromosome arm boundaries. The frequency of copy number gain is denoted in the top panel. Note the high frequency of 3q gain in the SqCC subtype, consistent with previous reports. Additional regions of copy-number difference are also clear, such as the more common gain of chromosome 2p in AC. The second panel (middle) shows the frequency of copy number loss. Common tumor suppressor gene loci such as chromosome 3p are common between AC and SqCC, but large differences exist in regions such as chromosome 4q. The significance of copy number disparity (inverse p-value corrected for multiple comparisons) between AC and SqCC subtypes is depicted in the third (bottom) panel. Solid black lines represent regions considered statistically different (p≤0.01) whereas grey lines are not.

Carcinomas of all types are known to harbor many DNA-level alterations linked, in part, to carcinogen exposure [25]. Indeed, tobacco smoke has been linked to the induction of not only DNA mutations, but also broad chromosomal instability [26]. Based on the differing exposure to tobacco carcinogens of cells in the central (SqCC) and peripheral (AC) airways, we sought first to determine whether global genomic instability was more prevalent in either of the two subtypes. We generated and compared whole genome copy number profiles for 261 NSCLC tumors 169 AC and 92 SqCC – by tiling-resolution array comparative genomic hybridization (CGH) (Sample Set #1, Table S1) [27], [28], [29], [30]. After hybridization, standard removal of systematic biases, and computational segmentation to identify regions of gain and loss, the number of gained, lost, and neutral probes was assessed for each tumor. The relative genomic instability observed in AC and SqCC groups was then compared (Figure 1a). The average number of altered probes (per sample) was compared between groups using the Mann-Whitney U-test. No significant differences between the two subtypes were found, consistent with previous work that showed similar DNA content across subtypes [31]. This analysis demonstrates that neither subtype has a proclivity for gain or loss of DNA. Therefore, observed differences in alteration frequency at a given locus can be attributed to sub-type specific selection of genes included within altered regions and not to different degrees of random genomic instability associated with tumor development.

### Disparate genomic landscapes characterize lung SqCC and AC

Although the NSCLC subtypes exhibit similar levels of genomic instability, if specific genetic pathways are involved in their differential development, differences in the genomic alterations selected during tumorigenesis should be present. To determine if genetic alterations unique to each NSCLC subtype exist, we looked for recurrent non-random regions of aberration in each group. Samples were grouped by subtype and probes were aggregated into regions based on similar copy number status. The frequency of alteration across autosomes was determined and compared between subtypes using the Fisher's exact test and the resulting *p*-values were corrected for multiple comparisons with a cut-off of ≤0.01 considered significant. In addition, we required regions to be altered in >20% of samples from a subtype group and a difference between groups of >10% to be considered “of interest”. Figure 1b displays the resulting genomic landscapes of AC and SqCC based on the frequency of gain and loss across the genome, and highlights the corresponding regions of difference between the subtypes that were identified.

This analysis revealed 294 regions of copy number disparity between SqCC and AC, 205 of which were SqCC-specific, whereas 89 were AC specific (Table S2). Although some regions overlapped, the character of the alteration (i.e. gain versus loss) was specific to an individual group. Since the alteration status between the subtypes differed strongly, we classed these as subtype-specific copy number alterations. In total, these alterations covered approximately 550 Mbp of the genome, ranging in size from large segments on chromosome arms (64.8 Mbp on 4q) to discrete peaks only kilobases in size (0.05 Mbp in multiple places).

Interestingly, copy number profiling of 20 preinvasive lung carcinoma *in situ* lesions, the assumed precursors to lung SqCC tumors, revealed that the vast majority (186/204, ∼92%) of SqCC-specific alterations are present at this stage of tumor development, suggesting that these events may commence early in SqCC tumorigenesis (Table S3). The remaining alterations that are present in the SqCC tumors and not found in the carcinoma *in situ* lesions may represent potential drivers of SqCC progression (Table S3, with potential target genes of these regions identified in Table S4, discussed below). Together, these findings support our hypothesis that the subtypes develop through different genetic pathways.

### Gene disruptions are selected in a subtype specific manner during NSCLC development

The discovery of DNA copy number disparities between NSCLC subtypes suggests that genes within these areas might be preferentially selected during tumorigenesis and thus, responsible for the differential development and pathological characteristics of the subtypes. To identify the potential target genes of these alterations, we integrated DNA copy number and gene expression levels [32]. Gene expression profiles were generated for a subset of tumors that were analyzed by array CGH (20 SqCC and 29 AC tumors, Sample Set #2, Table S1). We hypothesized that genes targeted by subtype-specific alterations would be different at the DNA level with matching differences at the gene expression level. Further, we also analyzed normal lung tissue to ensure that only genes differentially expressed in tumor tissues (relative to normal) remained as candidates, a characteristic consistent with a role in tumor development.

Genes located within each subtype-specific copy number alteration were identified and the expression levels compared between the SqCC and AC samples to determine those that were differentially expressed (p<0.001, after multiple testing correction). For SqCC, 4669 unique genes mapped to the subtype-specific copy number alterations (∼23 genes per region), and 797 (17%) of these were differentially expressed between subtypes in the anticipated direction. In AC, 2050 unique genes were located in subtype-specific copy number alterations (∼23 per region) and 171 (8%) were differentially expressed between subtypes in the anticipated direction.

Although some genes overlapped, their disruption patterns were specific to the individual cancer type, suggestive of opposing roles (oncogenic vs tumor suppressive) depending on cellular context. Thus, these genes were also considered to be subtype-specific targets. When combined, the SqCC and AC subtype-specific copy number regulated candidates represented 968 unique genes and showed a clear distinction in expression levels between the two subtypes.

**Figure 2:**
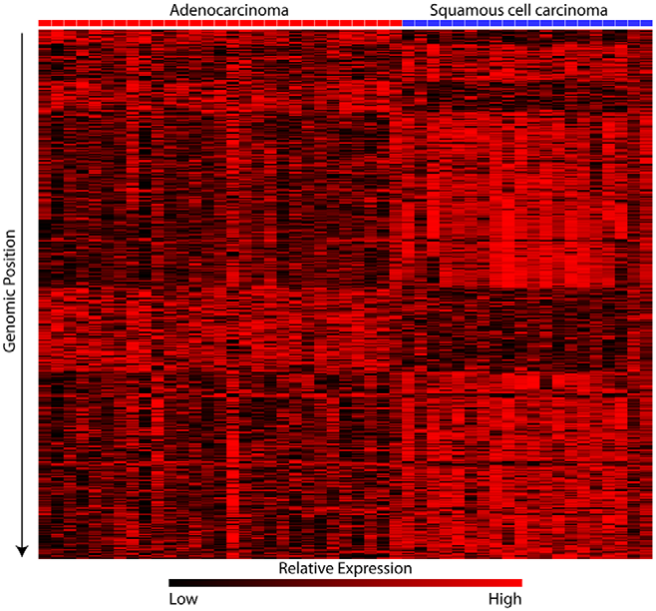
Differential expression as a result of subtype specific copy number alterations. Transformed absolute expression data for the 492 unique genes exhibiting disruption in expression levels as a result of copy number differences are displayed. In addition, these genes are up or down-regulated in the subtype which they are disrupted compared to normal lung tissue (see results). High-level expression is indicated by red while black indicates progressively lower levels of expression. The AC samples are indicated by red highlighting on the top of each column, while SqCC samples are indicated by blue highlighting. Each gene is sorted according to its chromosomal position. There is a clear distinction in the expression of these genes indicating their specific involvement in the subtypes.

In addition to demonstrating a relationship between expression and copy number alteration, a candidate subtype-specific gene was also required to be deregulated in cancer tissues relative to normal tissue [33]. We analyzed the expression levels of candidates in an independent panel of 53 SqCC and 58 AC lung tumors and 67 samples of exfoliated bronchial cells from cancer-free individuals (Sample Sets #3 and #4, Table S1). In total, 655 of the 797 SqCC-specific and 143 of the 171 AC-specific genes had corresponding probes on this array platform. These genes were compared between the respective cancer subtype and the normal bronchial cells in order to determine those that were significantly differentially expressed (p<0.001) in the direction predicted by the corresponding copy number alteration in which they were located. This analysis revealed that 447 (68%) of the SqCC-specific and 71 (49%) of the AC-specific genes were deregulated in cancerous tissues (492 unique gene alterations, Table S4). Since these genes met all three criteria for defining candidate subtype-specific, copy number alteration regulated targets as described above, we concluded they might represent the critical gene alterations driving the development of each subtype (Figure 2).

### Different oncogenic pathways are associated with the development of AC and SqCC

**Figure 3:**
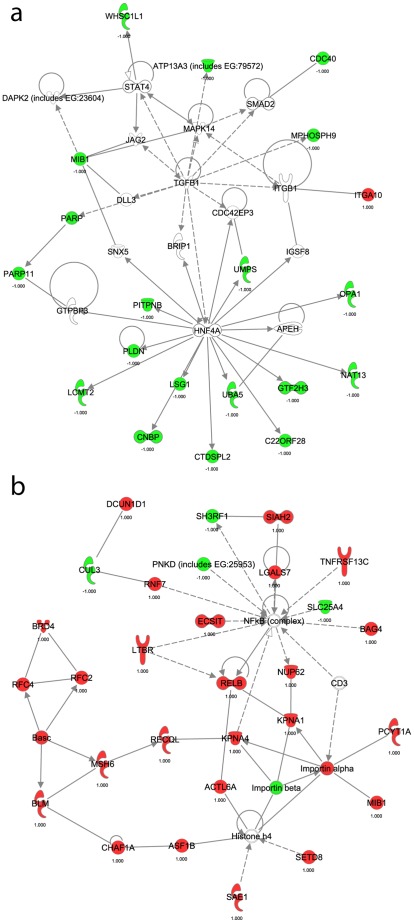
Gene networks involved in the development of SqCC and AC. Ingenuity Pathway Analysis was used to identify biologically related networks from the subtype specific genes deregulated by subtype-specific copy number alterations (see Methods). The top resultant gene networks for each subtype are displayed. a) AC network #1 of genes related to HNF4 signaling. b) SqCC network #1 displaying potential interactions between multiple histone regulating genes for both a) and b), solid lines denote direct interactions while dotted lines represent indirect interactions between the genes. Network components highlighted in red are upregulated in the corresponding subtype whereas those highlighted green are downregulated. Those not highlighted are used by the software to display relationships. Additional information about the genes and their interactions can be found at www.ingenuity.com. or within the discussion. In this diagram molecules are represented as such; corkscrews represent enzymes, y-shaped molecules are transmembrane receptors, thimble-shaped molecules are transporters, kinases are triangular, and circular molecules encompass all other gene products.

Cellular pathways and processes specifically disrupted in individual subtypes may reveal key oncogenic mechanisms driving the differential development of AC and SqCC. Thus, after identifying the genes responsible for the differences between the subtypes, we next wanted to investigate their biological functions. To discover subtype-related networks of biologically related genes we performed Ingenuity Pathway Analysis (IPA) of the 71 AC and 447 SqCC specific target genes (Figure 3, Table S5). SqCCs exhibited disruptions in gene networks that function in regulating DNA replication, recombination and repair, with additional roles in lymphoid tissue structure and development (Table S4). Genes involved in the top SqCC network were associated with the binding and modification of histone protein H4, as well as the regulation of the NFKB complex (Figure 3b). In contrast, the primary networks in AC displayed functions associated with cell-to-cell signaling, development, and drug metabolism (Table S5). The main AC-specific gene network was composed primarily of genes regulated by the transcription factor HNF4α (Figure 3a), whereas AC network 2 contained numerous genes controlled by TGFβ and TP53. The differential disruption of gene networks in AC and SqCC was further suggestive of distinct mechanisms of tumorigenesis for the subtypes.

### Global subtype variations in DNA methylation levels reflect differences in cells of origin

**Figure 4:**
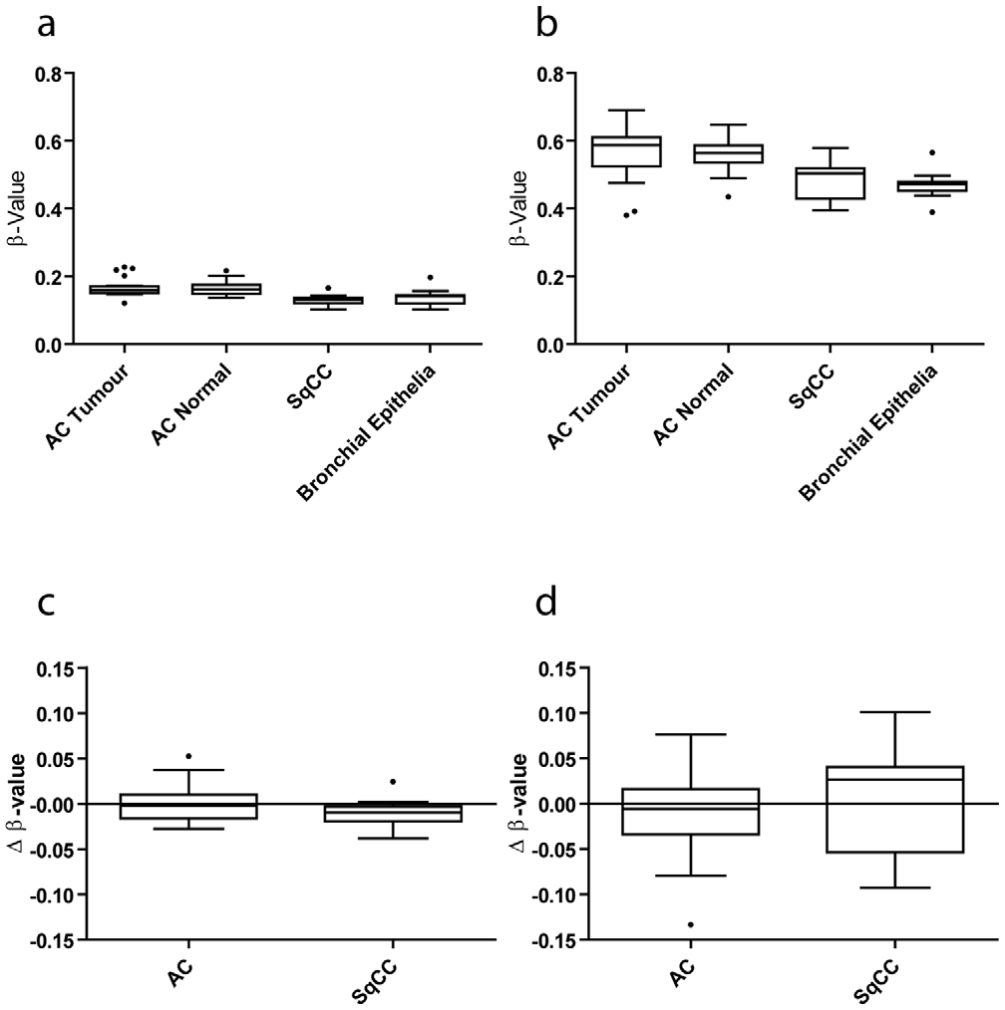
Global DNA methylation patterns of NSCLC tumors and associated normal tissues. Comparison of average DNA methylation levels between AC tumor, AC normal (histologically normal lung parenchyma), SqCC tumor, and bronchial epithelia. a) CpG island probe averages. The average of each of the profiles at probes located within CpG islands is plotted as a component within the box plot. In this panel, SqCC and bronchial epithelia samples appear to have slightly lower DNA methylation levels than the AC tumor and AC normal groups. b) Non-CpG island probe averages. The average of each of the profiles at probes not located within CpG islands is plotted as a component within the box plot. In this figure β-value is the level of methylation as defined by the methylated signal/total signal for each probe. In this panel, SqCC and bronchial epithelia are significantly lower in methylation level compared to the AC tumor or AC normal groups, indicating that outside of CpG islands, where the bulk of genomic methylation occurs, the central airway samples are more hypomethylated. c) Average differential methylation levels at CpG islands. The average differential is plotted for the 30 AC samples and the 13 SqCC samples. The two groups are very similar in their differential profile within CpG island probes. d) Average differential methylation levels at CpG sites not located within CpG islands. In this plot the average differential methylation level is plotted for probes that are not located within CpG islands. Again, the two groups are not significantly different by a Mann-Whitney U test.

Unlike the genome, which is identical for most normal cells in the body, the epigenome differs between tissue types [34], [35]. Similarly, cancer genomes exhibit global hypomethylation to varying degrees depending on the tissue of origin [36]. DNA methylation profiles are also influenced by mutational profiles within different cancer types, as DNA hyper- and hypomethylation alterations are also known to be related to tissue and genetic background [37] as well as smoking behavior [38]. Given the differing mutational spectra of the two NSCLC subtypes and their likely differing cells of origin, we investigated the overall DNA methylation level of 30 AC and 13 SqCC samples (Sample Set #4, Table S1). To enable comparisons of SqCC and AC tumors to appropriate matched normal cells, DNA methylation profiles were also generated for 30 non-malignant lung parenchyma samples (AC reference) and 18 histologically normal exfoliated bronchial epithelial cell samples (SqCC reference) from patients with NSCLC (Sample Set #1, Table S1). Analysis of 27,578 CpG dinucleotides probes within >13000 CpG islands shows that DNA methylation in the bronchial epithelia and the SqCC tumors was slightly lower than in the normal lung or AC tumors (Figure 4a). This is mirrored and exaggerated in the CpG dinucleotides outside of CpG islands, suggesting that the cells of the central airway are globally hypomethylated relative to the cells of the peripheral airways, whether cancerous or not (Figure 4b). In this case the two groups are significantly different when compared using a Mann-Whitney U test (*p*<0.0001).

To determine whether these trends were evident in tumor-specific epigenetic alterations, (i.e. those that exist within the tumor subtype when compared to an appropriate normal cell), we compared the differential methylation profiles of AC and SqCC tumors. These profiles were generated by subtracting the average normal DNA methylation profile for the references from each of the 30 AC and 13 SqCC tumors, respectively. Similar to the global assessment of copy number alterations (gain and loss), there were no significant differences between the AC differential profiles and the SqCC differential profiles (Figure 4c) in CpG island probes or non-CpG island probes (Figure 4d). Based on this, we again reasoned that any observed differences in hypermethylation or hypomethylation frequencies between the subtypes are likely to be due to subtype-specific selection of these alterations.

### Different epigenetic alterations are involved in the development of AC and SqCC subtypes

Although no differences in global methylation changes were observed between subtypes, the two subtypes may possess differential alteration frequencies at individual loci. To determine whether AC and SqCC tumors possess locus-specific differences in DNA methylation, we examined the frequencies of methylation alteration at gene-associated loci in both subtypes. Tumor DNA methylation levels were compared to the average of available normal reference tissue profiles. The frequency of probe hypermethylation and hypomethylation (tumor–normal |≥0.15) in AC and SqCC samples was compared using the Fisher's exact test. Following correction for multiple comparisons, 2708 probes corresponding to 2384 genes were found to be differentially methylated (p≤0.05). The SqCC group contained markedly more recurrently hyper- and hypomethylated loci than the AC group, similar to the disparity in the numbers of subtype-specific copy number-regulated genes observed in the analysis of genomic alterations. In fact, only 8% of the 2708 significant probes were more frequently altered in AC, the rest being more commonly hyper- or hypomethylated in SqCC.

To further refine the list of differentially methylated genes to those whose gene expression reflects levels expected based on their epigenetic alteration, we assessed the 2384 genes with differential methylation for differential expression between the subtypes, as well as differential expression from normal tissues, using a corrected p-value threshold of *p*<0.05. 32 AC candidate genes and 297 SqCC genes met these stringent criteria and were further analyzed as subtype-specific epigenetically regulated genes (Table S6).

### Epigenetically regulated genes complement genetically regulated genes

**Figure 5:**
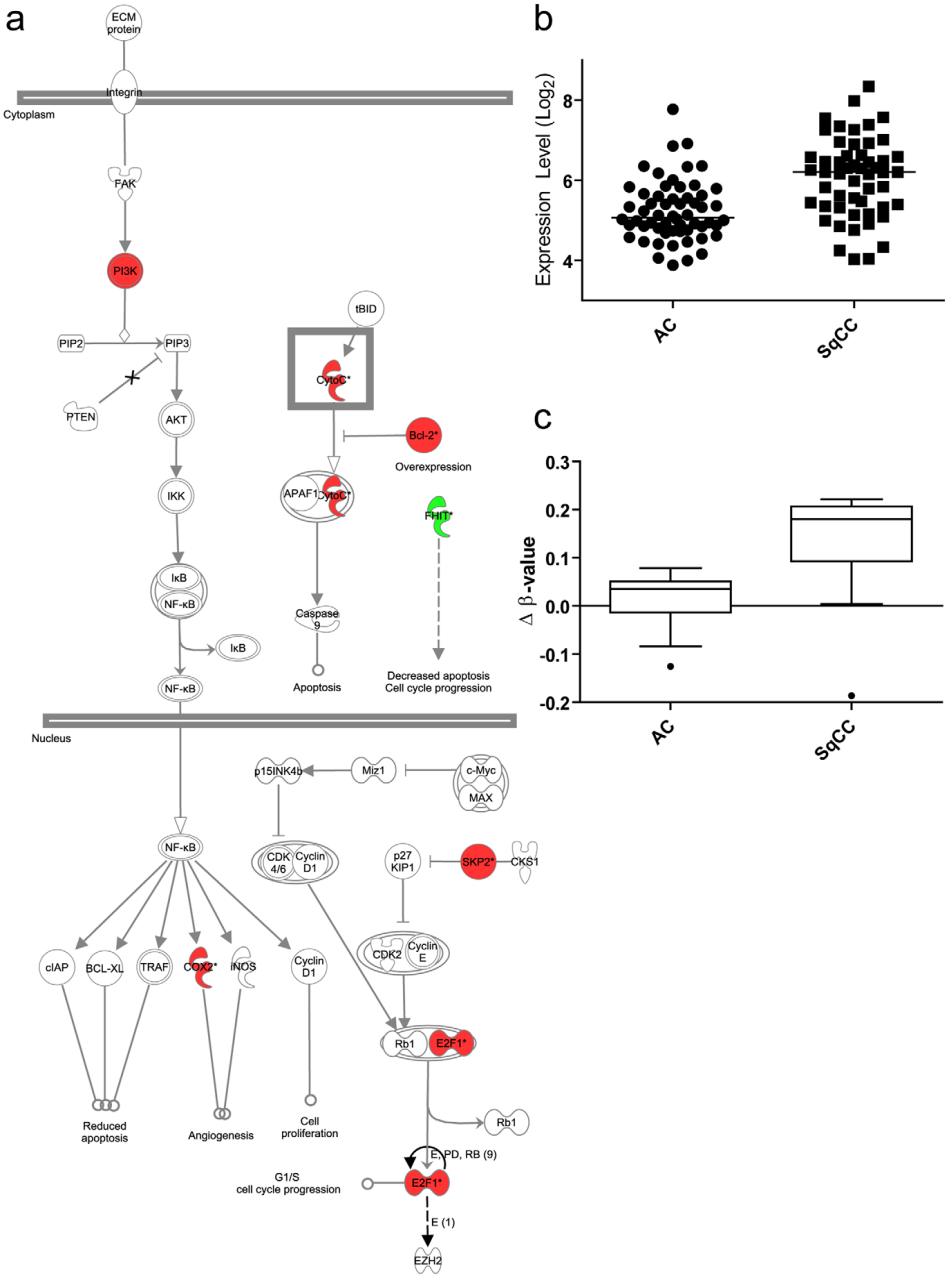
Epigenetically altered SqCC genes are significantly enriched for SCLC signaling. a) SCLC signaling components altered by DNA methylation in SqCC. In this schematic of the SCLC signaling pathway, genes that are hypomethylated and overexpressed are shown in red, and those that are hypermethylated and underexpressed are shown in green. Components at all levels of the pathway are affected, including the transcription factor *E2F1*, which drives the expression of the oncogenic polycomb group member *EZH2*. b) *EZH2* expression in 58 AC tumors and 53 SqCC tumors. *EZH2* expression was assessed in an external dataset, and it was found to be higher, as predicted, in SqCC tumors compared to AC tumors using a Mann-Whitney U test (p<0.0001). c) *FHIT* differential methylation levels in SqCC and AC tumors. *FHIT* was shown to be deregulated by both deletion and hypermethylation in a manner that was specific to SqCC tumors. Show here are the differential DNA methylation levels for 30 AC tumors and 13 SqCC tumors. The SqCC tumors are hypermethylated to a much higher degree than the AC tumors, consistent with previous published findings.

To determine whether the 32 AC-specific and 297 SqCC-specific epigenetically-regulated genes carried out functions similar to those subtype-specific genes discovered by the DNA copy number analysis described above, pathway disruption analysis was performed. This revealed that the most significant epigenetically-regulated gene network in AC is involved in cell cycle, cell death, and cellular development (Table S7). This is partly in contrast to the top AC network of copy number regulated genes, which similarly have functions associated with tissue development, but also possess cell signaling and hematological system function in common (Table S4). The overall degree of similarity between AC-specific genes that are genetically or epigenetically regulated is quite small, likely due to the low number of AC-specific genes identified (potential reasons for this are discussed below). In contrast, the SqCC gene networks in both analyses are very similar. For example, DNA replication, recombination and repair are highly featured functions of genes identified by both DNA copy number and DNA methylation analyses of SqCC (Tables S5 and S7, respectively). Additionally, genes involved in immunological disease and lymphoid tissue structure and development were prominent. Of particular interest was the enrichment of aberrantly methylated genes in the small cell lung cancer signaling pathway (comprised of genes known to deregulated in small cell lung cancer as annotated by Ingenuity) (Figure 5a). This was the most significantly enriched canonical pathway in either subtype that was affected by DNA methylation alterations and it is of interest because both of these lung cancers (SCLC and SqCC) arise in the central airways with similar exposure to cigarette smoke carcinogens. *E2F1* is among the hypomethylated and overexpressed genes represented in this pathway, and is known to be overexpressed in SCLC and to drive expression of *EZH2,* which is also overexpressed in SCLC [39], [40]. To explore this pathway further, we investigated whether *EZH2* was more highly expressed in SqCC than AC tumors (as a consequence of differential *E2F1* expression). As expected, we found that *EZH2* was expressed at a significantly higher level in SqCC tumors than AC tumors, demonstrating the biological consequence of *E2F1* disruption (Figure 5b). The differential expression of *EZH2* in the two subtypes is significant, given the numerous differences in aberrant DNA methylation observed between subtypes; this could reflect the key role of EZH2 in the polycomb group, a protein complex involved in DNA methylation [41].

DNA copy number and DNA methylation data are complementary from a gene-specific perspective as well. This is highlighted by seven genes (*ATP2C1*, *PCYT1A*, *ZWILCH*, *CENTB2*, *BAG4*, *PARP11* and *CSDA*, Table S8) that are disrupted by gene-dosage in one subtype and DNA methylation in the other. *PARP11* is one such example of differential activation/inactivation by DNA copy number and DNA methylation, which is discussed further below.

### Concerted genetic and epigenetic disruption of subtype-specific genes

In order to determine if both DNA copy number and DNA methylation aberrations simultaneously disrupted any genes, we combined the subtype-specific gene lists derived using the two analytical approaches described above (Table S9). Combining the 71 AC genes identified through their association with DNA copy number alteration and the 32 genes associated with DNA methylation aberrations did not yield any overlapping genes. This result was not surprising given the observed lack of similarity at the level of function/network analysis. In SqCC however, combining the 447 copy-number associated genes with the 297 DNA methylation genes yielded overlap of 38 genes (Table S9). These genes exhibit frequent concurrent genetic, epigenetic, and subsequent gene-expression alterations that discriminate them from AC tumors. Notably, the well-known 3p tumor suppressor gene (TSG) *FHIT* was among these genes. The differential methylation levels of *FHIT* are shown in Figure 5c. Loss of *FHIT* expression is associated with smoking and is more frequent in SqCC tumors than AC tumors, consistent with our data [42], [43], [44]. Hypermethylation of this gene has also been investigated as a potential biomarker for centrally-occurring lung cancers [45], [46]. Concerted genetic and epigenetic disruption was not limited to hypermethylation/loss however, as numerous genes displayed hypomethylation coupled with increased copy number and gene expression in SqCC. For example, *BRF2*, which we recently identified as a lineage specific oncogene in lung SqCC, was deregulated in this manner, highlighting its importance to the development of this lung cancer subtype [47]. Furthermore, in addition to those genes previously associated with lung cancer, TSGs and oncogenes known to be deregulated in other cancer types–such as *PRDM2* and *SIAH2*, respectively – were also altered at the genetic, epigenetic and gene expression levels. Such multidimensional disruption is indicative of strong selective pressures for gene silencing/activation during tumorigenesis, suggesting that these genes may play a pivotal role in SqCC.

### Subtype specific genes are responsible for AC and SqCC phenotypes

**Figure 6:**
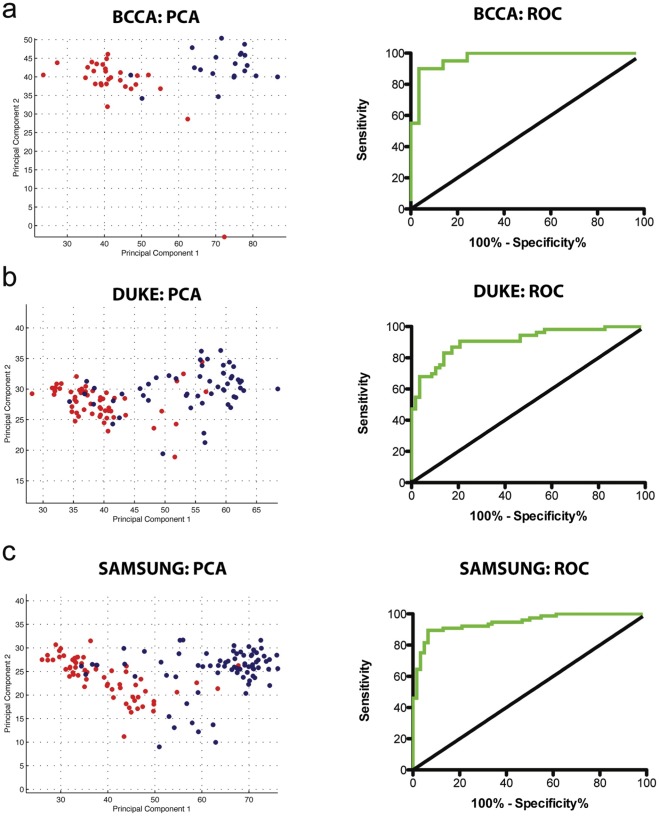
Subtype specific genes explain AC and SqCC phenotypes. Principal components analysis was performed utilizing all genes demonstrating expression differences between the subtypes as a result of genetic and/or epigenetic alterations using: A) Data generated for 49 NSCLC tumors (29 AC, 20 SqCC) as part of this study which was used in gene discovery; B) Publically available data from 111 NSCLC tumors (58 AC and 53 SqCC, Dataset #3-Duke, Table S1) used as test set #1; C) Publically available data from 138 NSCLC tumors (62 AC and 76 SqCC, Dataset #6-Samsung, Table S1) used as test set #2. Red circles indicate AC samples, while the blue circles indicate SqCC samples. Strong separation of the AC and SqCC tumors along principal component 1 in all sets demonstrates the contribution of these genes to the differential phenotypes. On the right are the respective ROC curves for each dataset using the respective principle component 1 values for each sample. AUC values of 0.9690, 0.9076 and 0.9442 for A), B) and C), respectively, suggest that the gene expression signature is an extremely good discriminator of the subtypes.

Next, we aimed to confirm that the genes differentially disrupted at the genomic and epigenomic level are responsible for the different biological characteristics of AC and SqCC. Since they are regulated by subtype specific alterations, we hypothesized that the expression levels of these genes should be able to accurately segregate NSCLC tumors into distinct AC and SqCC groups. As predicted, when using the expression values for the 49 NSCLC tumors from our data set, principle component analysis with the 778 unique genetically and/or epigenetically deregulated genes clearly delineated distinct subtype specific clusters (Figure 6a). A receiver operating characteristic (ROC) area under the curve (AUC) value of 0.9690 (P<0.0001) confirmed that principle component 1 was a strong discriminator of the subtypes (Figure 6a). This was not surprising as the genes were uncovered based on differences between the subtypes using this same set of samples. Therefore, to further confirm the role of the genes in subtype development, we applied the same analysis to two independent sample sets generated by different institutions. The first consisted of 111 (58 AC and 53 SqCC, Sample Set #3, Table S1) and the second of 138 (62 AC and 76 SqCC, Sample Set #6, Table S1) clinical lung tumors [48]. Strikingly, this analysis was also able to separate the AC and SqCC samples with a great deal of accuracy (ROC AUC values of 0.9076 and 0.9442, P<0.0001, respectively) (Figure 6b and c). Validation in these large, independent panels of NSCLC tumors from separate institutions provides further evidence that the genes regulated by subtype specific genomic and epigenomic disruptions are responsible for driving the differential development of AC and SqCC. Furthermore, our results highlight the impact of this novel integrative genome, epigenome and transcriptome analysis in identifying robust target genes that can be used as biomarkers of disease.

### Subtype-specific genetic differences are translated to the protein level

**Figure 7:**
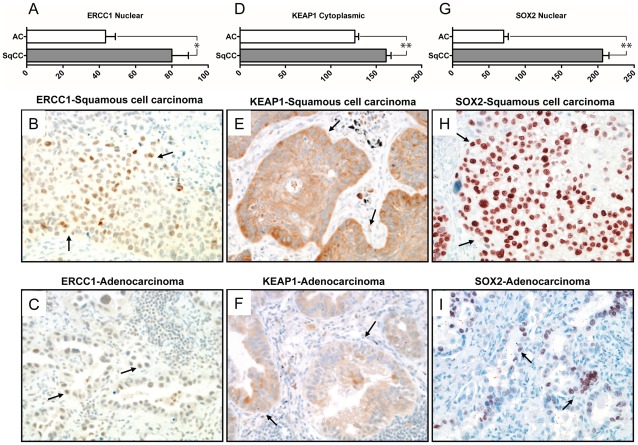
Subtype-specific genomic differences are reflected at the protein level. Immunohistochemical analysis of protein levels for ERCC1 (A–C), KEAP1 (D–F) and SOX2 (G–I) in squamous and adenocarcinoma lung tumors. Average immunohistochemical protein expression levels for each subtype are plotted ± SEM of each group. Representative microphotograps showing tumoral cells (arrows) with higher levels of immunohistochemistry expression of nuclear ERCC1 (*B* and *C*), cytoplasmic KEAP1 (*E* and *F*) and nuclear SOX2 (*H* and *I*) in squamous cell carcinomas (*B, E and H*) compared to lung adenocarcinomas (*C, F, and I*). Images are of samples reflecting the average protein expression for each group (ERCC1: SqCC  =  ∼80, AC  =  ∼43; KEAP1: SqCC  =  ∼161, AC  =  ∼126; SOX2: SqCC  =  ∼207, AC  =  ∼70). Magnification 200x. * and **  =  p<0.001 and p<0.0001, two-tailed unpaired t test with Welch's correction, respectively.

In order to confirm that the genome and transcriptome differences between the subtypes affect the relative protein levels of the identified genes, we performed immunohistochemical (IHC) analysis on a large, independent panel of >200 lung tumors. Protein levels for three subtype specific genes with available antibodies validated for IHC were analyzed: ERCC1 (inactivated in AC), KEAP1 (inactivated in AC) and SOX2 (activated in SqCC) (Figure 7). Average protein levels for all three genes were significantly different between the subtypes in the direction predicted by the integrative genetic and epigenetic analysis (Figure 7a, d, and g). The average immunohistochemical nuclear ERCC1 expression was much lower in AC tumors (43.45±5.389, N = 175) compared to SqCC tumors (79.99±9.095, N = 106, two-tailed p<0.001, unpaired t test with Welch's correction) consistent with this gene being inactivated specifically in AC. Similar results were seen for cytoplasmic levels of KEAP1, which is also inactivated specifically in AC (AC: 126.5±4.179, N = 184; SqCC: 160.9±5.401, N = 110, two-tailed p<0.0001, unpaired t test with Welch's correction). Conversely, nuclear levels of SOX2, which is activated specifically in SqCC, were significantly higher in this subtype (206.5±8.839, N = 106) than in AC (70.39±6.342, N = 170, two-tailed p<0.0001, unpaired t test with Welch's correction). Together, these data demonstrate that the genomic, epigenomic and gene transcription differences between the subtypes are translated to the protein level, providing more credence to the hypothesis that these changes have a functional consequence on the phenotypes of AC and SqCC.

### Subtype-specific genes are associated with distinct clinical characteristics in AC and SqCC

**Figure 8:**
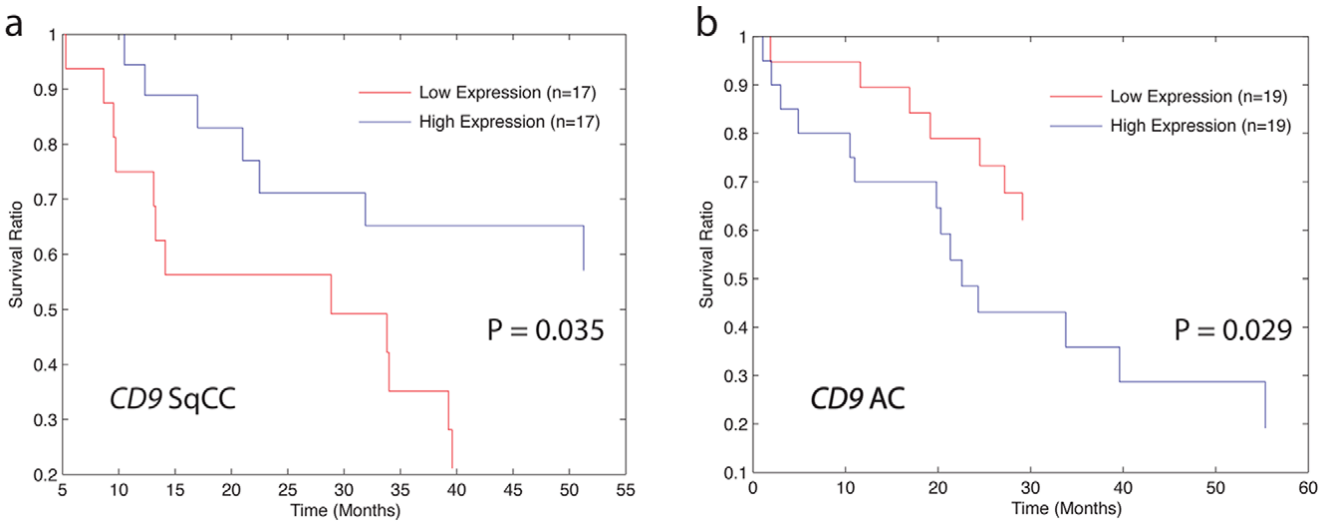
*CD9* alteration and survival is different in AC and SqCC tumors. a) Low *CD9* levels are associated with poor prognosis in SqCC. The prognostic value of *CD9* expression levels was evaluated in 53 SqCC tumors. Survival of the 1/3 lowest *CD9* expressers is shown in red, and the top 1/3 is shown in blue. In this case, low expression of *CD9* is significantly associated with poor prognosis when using a Mantel-Cox log test (*p* = 0.035). b) High *CD9* levels are associated with poor prognosis in AC. The prognostic value of *CD9* expression levels was evaluated in 58 AC tumors. Survival of the 1/3 lowest *CD9* expressers is shown in red, and the top 1/3 is shown in blue. In this case, low expression of *CD9* is significantly associated with poor prognosis when using a Mantel-Cox log test (*p* = 0.029).

We next aimed to determine the influence of the subtype specific genes on the clinical characteristics of AC and SqCC. Since these genes are responsible for defining the distinct biology of these diseases, we reasoned that their expression should only correlate with specific clinical features in one subtype and not the other subtype or NSCLC (AC + SqCC) in general. To test this, we determined the survival associations using a Mantel-Cox log rank test for each of the 778 subtype specific genes in AC, SqCC and NSCLC in the dataset with overall survival information available (Sample Set #3, Table S1). Collectively, this analysis revealed 131 AC and 46 SqCC specific genes that had significant (P<0.05) associations with overall survival (Table S10). Remarkably, the associations were completely specific to an individual subtype as no genes were correlated with survival in the same manner across both subtypes. Six genes (*DSG2*, *PLAC2*, *ATP9A*, *TPM4*, *CD9* and *PSMD11*) were significantly associated with survival in both subtypes; however, they displayed a completely opposite pattern in terms of survival with low expression associated with poor survival in one subtype and high expression with poor survival in the other (Figure 8). Thus, although associated with survival in both subtypes, the genes exhibit distinct subtype-specific associations. Interestingly, low levels of *CD9* expression have been previously implicated in the poor prognosis of lung cancer patients [49]. However, we now show that this association is subtype specific with low levels of *CD9* correlated with poor prognosis in SqCC and high levels with poor prognosis in AC (Figure 8). Importantly, only eight genes that were associated with survival in one of the subtypes were also significant when analyzing NSCLC as a whole, providing further evidence to the importance of treating the subtypes as separate disease entities. Together, these findings underscore the potential clinical relevance of subtype specific alterations.

### Defining putative treatment strategies tailored to lung cancer subtypes using *in silico* screening of candidate therapeutic compounds

Lastly, after defining and validating our AC and SqCC specific cancer genes, we applied these findings to define potential treatment strategies tailored to each lung cancer subtype. To do this, we queried the Connectivity Map (CMAP) database using our subtype specific genes to identify compounds that could “reverse” the expression direction of each signature. The CMAP consists of thousands of gene expression profiles from different cancer cell lines treated with a vast collection of small molecules [50]. By comparing the subtype specific signatures of up and down regulated genes with preexisting small molecule response signatures in the database, the program can identify small molecules whose effects on gene expression changes are positively or negatively correlated. Negative correlation scores imply that the matched molecules have a mode of action that can reverse the expression direction of query genes, and therefore serve as potential therapeutic compounds. Using this *in silico* screening approach, we identified numerous instances (cell line/treatment combination) that were significantly correlated with both the AC and SqCC specific gene signatures identified in our study (Table S11). Remarkably, SqCC had an expression signature that was negatively correlated with multiple HDAC and PI3K/mTOR instances including trichostatin A, vorinostat (also known as SAHA) and MS-275 (all HDAC inhibitors) and LY-294002, quinostatin, sirolimus (also known as Rapamycin) and wortmannin (all PI3K/mTOR inhibitors). These findings were interesting for two reasons, firstly as the alteration of histone modifying enzymes was the major network disrupted in SqCC (see above). Secondly, the relevance of these epigenetically-targeted drugs is pertinent as we identified concerted disruption of PRC2 components responsible for *de novo* methylation as well. In addition, *PIK3CA* activation (mutation and/or amplification) is known to occur more frequently in SqCC than AC [51] and many downstream components of this pathway were also altered specifically in SqCC (Table S4). The CMAP analysis for AC, on the other hand, was not very informative, as none of the negatively correlated molecules shared the same functions (Table S10). This may be a byproduct of the heterogeneity between AC tumors (discussed below).

**Figure 9:**
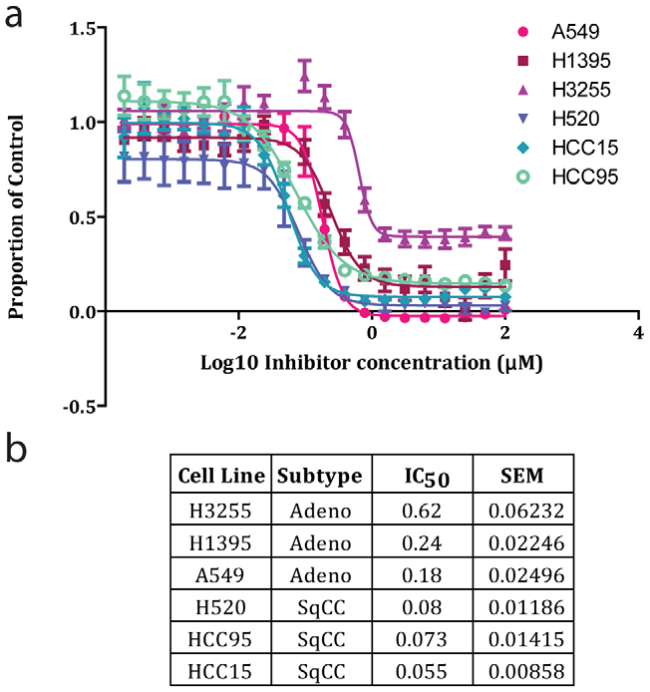
SqCC cell lines are more sensitive to the HDAC inhibitor Trichostatin A than AC cell lines. a) Dose-response analysis of Trichostatin A on the relative viability of three AC (A549, H3255 and H1395) and three SqCC (H520, HCC15 and HCC15) cell lines. Each curve was generated from the average data points from four separate experiments. b) Table with the average IC_50_ and SEM for each cell line tested derived from four separate experiments.

To confirm the results from the CMAP analysis, we treated a panel of six NSCLC cell lines (three AC and three SqCC) with the HDAC inhibitor Trichostatin A, which was the most significantly negative correlated HDAC inhibitor from the SqCC analysis. Importantly, we selected the available cell lines that best represented their respective clinical tumor subtypes by performing principle component analysis with the subtype-specific genes using publically available gene expression profiles for a large panel of NSCLC cell lines (Figure S1). As predicted by the *in silico* analysis, SqCC cell lines (average IC_50_ = 69 nM) were, on average, five times more sensitive to Trichostatin A than AC cell lines (average IC_50_ = 346 nM), a statistically significant difference (P = 0.05, Mann-Whitney U Test) (Figure 9).

## Discussion

The emergence of tumor cells from normal precursors is thought to involve a complex interplay between genetics and cell lineage [8]. Due to the different cell types involved as well as the attributes of an individual cell's local environment or niche, it is logical to assume different mechanisms are required in tumorigenesis for each lung cancer subtype. Cell lineage may also have a dramatic effect on the manifestation of genetic/epigenetic alterations during the development of each lung cancer subtype as only those promoting a malignant phenotype in the specific cellular context will be selected and maintained [8]. Previous studies suggest that distinct patterns of DNA alteration exist for AC and SqCC; however, the specific genes responsible for the different tumor phenotypes are largely unknown [20], [21], [22], [52].

In this study, we provide the first comprehensive investigation of the key genetic and epigenetic alterations distinguishing AC and SqCC lung tumors. We achieved this by integrating whole-genome DNA copy number, DNA methylation, and gene expression data to identify genes altered in a subtype-specific manner. These genes are associated with distinct gene networks in each lung cancer subtype, flagging distinct signaling pathways as contributing to tumorigenesis. We also found subtype-type specific changes to be correlated with clinical outcomes and highlight putative treatment strategies based on the subtype specific molecular signatures.

The 294 subtype-specific copy number alterations detected in this study demonstrate that different genetic pathways are involved in the pathogenesis of AC and SqCC. Importantly, previously identified lineage specific oncogenes including *SOX2* and *BRF2* were identified, validating our approach [17], [47]. Although some of the regions and genes have previously been shown to be important in NSCLC development, our findings suggest their newfound importance to a specific lung cancer subtype. For example, previously identified oncogenes such as *NOTCH3* and *FOXM1* were overexpressed through increased gene dosage specifically in SqCC while the tumor suppressor *KEAP1* was deleted and underexpressed specifically in AC [53], [54], [55]. This is the first report suggesting that these previously established lung cancer-associated genes are actually involved in subtype-specific tumorigenesis.

A broader gene network-based analysis of the copy number-regulated genes revealed additional insights into the differential oncogenic mechanisms driving the pathogenesis of AC and SqCC. The top SqCC gene network was mainly associated with DNA replication, recombination and repair. In addition to these functions, histone modification genes were represented as well. Histones are fundamental building blocks of eukaryotic chromatin and are involved in myriad cellular processes, including replication, repair, recombination and chromosome segregation [56], [57], [58]. Recently, global alterations of histone modification patterns have been reported in human cancers [59]. Our data suggest that direct deregulation of histone modification enzymes including *ASF1B*, *PRMT1*, *SAE1*, *SET8*, *CHAF1A* and *UHRF1* may drive this phenomenon and play a key role during the development of lung SqCC. As histone modifications also play an essential role in DNA replication, there may be a synergistic effect between the histone modifying genes and replication/recombination associated genes that contribute to tumor development. Interestingly, histone modification alterations occur more frequently in lung SqCC than AC, consistent with our findings [60].

The gene network detected as perturbed in AC subtype tumors contained genes mainly involved in regulating tissue development and cell-to-cell signaling and known to be targeted by the transcription factor HNF4α. HNF4α regulates a large set of genes in a cell-specific manner and is necessary for cell differentiation and maintenance of a differentiated epithelial phenotype [61]. In other carcinomas, deregulation of *HNF4α* leads to increased cellular proliferation, progression and dedifferentiation [62], [63], [64], [65], [66]. This suggests that *HNF4α* may act as a tumor suppressor in epithelial carcinogenesis [61]. Interestingly, although *HNF4α* was not affected, we found that numerous downstream targets of this gene are downregulated specifically in AC. Thus, this may have the same net affect as inactivation of *HNF4α* itself and lead to increased cellular proliferation during AC tumorigenesis.

Concerted alterations to gene networks and pathways are not a feature that is limited to copy-number regulated genes. Indeed, we found that coupling subtype-specific DNA methylation profiles with matched gene expression alterations implicated numerous canonical signaling pathways in the differential development of SqCC and AC tumors. The enrichment of small-cell lung cancer signaling pathway members within the epigenetically altered SqCC genes was of particular interest. For example, one of the deregulated components of this pathway, the transcription factor *E2F1,* was found to exhibit SqCC-specific hypomethylation and overexpression. *E2F1* is upregulated in SCLC tumors [40], which suppresses apoptosis and induces expression of *EZH2*, an oncogenic polycomb histone-methyltransferase [39]. The relevance of this pathway in SqCC tumors is strengthened by our observation that *EZH2* expression is significantly higher in SqCC than AC (Figure 5). This is particularly interesting given the potential dual role of *EZH2* in different cancer types [67]. The disruption of the polycomb group (preferentially in SqCC) is relevant because we have also identified SqCC-specific deregulation of numerous histone-modifying enzymes by DNA copy number alterations.

In addition to the deregulation of histone modifying genes by DNA copy number and DNA methylation alterations, we have uncovered evidence of global SqCC-specific epigenetic disruption. Our analysis of global DNA methylation levels in AC and SqCC tumors showed that SqCC tumors were more hypomethylated overall, suggesting that the epigenetic machinery is highly deregulated in SqCC (Figure 4). There is precedent for this finding, as altered global methylation is thought to be a consequence of exposure to the carcinogens found in tobacco smoke [37], [38], [68]. Global hypomethylation, such as that caused by cigarette smoke, is also known to be associated with chromosomal instability. Although we did not observe any difference in the percentage of AC or SqCC genomes that were altered by copy number, we did identify a greater number of recurrent copy number alterations in the SqCC subtype. This may be indicative of similar selective pressures in the SqCC tumors that facilitate the development of recurrent alterations, whereas those in AC may be more diverse, leading to greater heterogeneity.

Concerted DNA copy number and DNA methylation alterations yield insight into tumor biology as well. We show hypermethylation and deletion of *FHIT* to be a SqCC-specific event, confirming earlier studies describing inactivation of the gene at a higher frequency in SqCC than AC tumors [42], [46], [69], [70]. While there were relatively few genes that were simultaneously activated/inactivated in SqCC by DNA copy number and methylation alterations (32), there was no overlap seen in AC. In fact, compared to SqCC tumors, AC tumors possessed fewer subtype-specific alterations linked to both DNA copy number and DNA methylation. The reason for this is not clear, but it is possible that AC tumors have higher levels of cellular and/or genetic heterogeneity than SqCC tumors. Heterogeneity of patient clinical-characteristics may also contribute to this, as lung cancer in non-smokers are more likely to appear as AC tumors, and cigarette smoke may play a role in contributing to specific genetic or epigenetic alterations [71], [72]. Nevertheless, although a high proportion of our AC tumors were from never smokers (22.5%), no significant differences in copy number were identified between AC tumors from ever and never smokers (data not shown), suggesting that this is not a confounding factor in our analysis.

The specific alterations selected during the development of each subtype may also play a role in the clinical management of disease, such as influencing treatment outcomes. Indeed, genes already known to influence NSCLC response to conventional chemotherapy were deregulated in a subtype-specific manner. For example, the finding that *ERCC1* disruption was subtype-specific is significant. *ERCC1* is a nucleotide excision repair gene which repairs DNA adducts and lesions induced by smoking-related carcinogens [73]. As such, low expression levels of *ERCC1* have been implicated in lung cancer susceptibility [74] and tumorigenesis, whereas high expression levels are associated with favorable overall prognosis [73]. However, since *ERCC1* is also involved in the repair of cisplatin-induced DNA adducts in cancer cells, high expression levels increase resistance to platinum-based chemotherapies [75], [76], while low expression leads to drug sensitivity [77]. Underscoring the relevance of this finding are the results of recent clinical trials that have described a significantly better outcome for patients who received adjuvant cisplatin-based combination chemotherapy if their resected tumors expressed low levels of *ERCC1* [73], [75]. Our finding that this gene is inactivated specifically in AC tumors has major clinical consequences in terms of guiding disease management and treatment strategies in order to define appropriate treatment regimens for patients. This is consistent with a previous report demonstrating the subtype specificity of *ERCC1* expression levels in NSCLC, and further highlights how biological differences between AC and SqCC may influence patient response to therapy [78].

Importantly, numerous genomic regions showed opposite patterns of alteration in each lung cancer subtype. For example, a discrete alteration spanning 2.4 Mbp on chromosome bands 8p12-p11.23 was commonly gained in SqCC and lost in AC, implying that genes in these regions may play opposite roles during the development of the individual NSCLC subtypes, acting as TSGs in AC and as oncogenes in SqCC. Such diametric alteration is seen when including epigenomic alterations as well. This is the case for *PARP11*, which is upregulated in SqCC by DNA hypomethylation and downregulated in AC by copy number loss (Table S8). This information will become particularly important as targeted therapeutic strategies based around these genes develop. The development of MEK inhibitors highlights this point [72]: since activated MEK1 and MEK2 phosphorylate and activate ERK (MAPK1), the differential deregulation of *MAPK1* in AC (inactivated) and SqCC (activated) tumors may be an important consideration in determining the efficacy of this treatment against lung cancer subtypes [79].

Similarly, numerous studies have aimed to identify genes associated with prognosis in NSCLC in order to better determine patient outcome [80]. Our data suggest that these relationships may be subtype-specific as well (Table S10). Importantly, we discovered that specific genes may be indicative of totally different clinical outcomes depending on which subtype they are disrupted in. For example, *CD9* was gain/overexpressed in SqCC and high expression of this gene correlated with favorable survival in this subtype as well (Figure 7). However, the opposite was true in AC, which displayed copy number loss and underexpression; low expression was associated with good survival and high expression with poor survival. Together, these results indicate that the genes involved in defining clinical characteristics are largely exclusive to individual NSCLC subtypes and influenced by the acquisition of distinct genetic alterations during tumor development. In addition, this underlines the importance of separating AC and SqCC when assessing genes involved in predicting patient prognosis and other clinical outcomes.

Furthermore, in order to demonstrate how these findings can be used to define treatment strategies tailored to the individual lung cancer subtypes, we performed CMAP analysis using our AC and SqCC specific gene signatures to identify compounds that can potentially reverse the expression of these genes. Although the results for AC were uninformative, the SqCC CMAP analysis identified numerous HDAC and PI3K/mTOR inhibitors as compounds that could potentially induce a gene expression signature negatively correlated with that associated with SqCC (Table S11). The HDAC inhibitor result was remarkable as the alteration of histone modifying enzymes was the most prominent network disrupted in this subtype, providing a biological basis for this finding. Furthermore, cancer cells with elevated activity of E2F1 have been shown to be highly susceptible to HDAC inhibitor induced cell death and more recently HDAC inhibitors such as SAHA have been shown to suppress the activity of EZH2 [81], [82]. As E2F1 and EZH2 are both upregulated in SqCC (Figure 5), this data suggests that treatment with HDAC inhibitors, in conjunction with standard chemotherapy, could be a promising avenue for disease treatment. In addition, since *PIK3CA* activation (mutation and/or amplification) is known to occur more frequently in SqCC than AC the finding of multiple PI3K/mTOR inhibitors as potential therapeutics for SqCC is also logical [51], [75]. Together, this data demonstrates the potential to use information about the underlying molecular biology of the cancer subtypes to make informed decisions about clinical management strategies and suggests that HDAC and PIK3/mTOR inhibitors, in combination with current treatment regimes, may provide a novel treatment tailored to lung SqCC.

Lastly, it is important to note that although they display broadly unifying characteristics, AC and SqCC themselves are very heterogeneous tumor types, with many molecular, pathologic and clinical subtypes [83], [84]. We suggest that our analysis has revealed the common initiating molecular changes for AC and SqCC, which may be followed by secondary driver mutations that cause the subsequent heterogeneity seen in advanced tumors. This is supported by the fact that we identified SqCC specific alterations in preinvasive CIS lesions, suggesting that these alterations commence early in tumor development (Table S3). By identifying these “root” changes, one may be able to utilize type specific therapies either in combination with, or followed by, individualized therapies that target the secondary alterations to achieve a more complete antitumor response.

### Conclusions

Fundamental discrepancies in tumor biology may be a primary factor determining the differential outcomes of lung cancer patients. Biological differences that segregate with cell lineages may also lead to differences in response to therapies [85]. Therefore, tumor cell lineage may be an important consideration when selecting and developing therapeutic approaches for lung cancer. An example of this is already in common practice as SCLC and NSCLC are treated separately due to the observation that cancers of the former lineage tend to be much more responsive to initial treatment with conventional cytotoxic agents. In contrast, no clinical distinction is made between the different subtypes of NSCLC and stage is the primary factor that determine treatment options. Our high-resolution integrative analysis of NSCLC genomes and epigenomes delineated novel tumor subtype-specific genetic and epigenetic alterations responsible for driving the differential pathogenesis and phenotypes of AC and SqCC. The specific genes and networks identified in this study provide essential starting insights for elucidating mechanisms of tumor differentiation and developing tailored therapeutics for lung cancer treatment. More generally, our results confirm at the molecular level that these lung cancer subtypes are distinct disease entities. When designing new treatment strategies and testing new drugs in clinical trials, these subtype differences as well as the biological pathways should be taken into account.

## Materials and Methods

### Ethics Statement

All patient samples were collected under informed, written patient consent and anonymized as approved by the University of British Columbia–British Columbia Cancer Agency Research Ethics Board (REB number H04-60060).

### DNA samples

Formalin-fixed, paraffin embedded (FFPE) and fresh-frozen tissues were collected from St. Paul's Hospital, Vancouver General Hospital and Princess Margaret Hospital following approval by the Research Ethics Boards. Hematoxylin and eosin stained sections for each sample were graded by a lung pathologist for use in selecting regions for microdissection. DNA was isolated using standard procedure with proteinase K digestion followed by phenol-chloroform extraction as previously described [86]. Patient information is located in Table 1.

**Table 1:** Sample set clinical characteristics.

		AC (n = 169)	SqCC (n = 92)
Stage*	I	76 (44.9%)	32 (34.7%)
	II	40 (23.6%)	32 (34.7%)
	III	22 (13%)	14 (15.2%)
	IV	27 (16.0%)	10 (10.9%)
	n/a	4 (2.4%)	4 (4.4%)
Sex	Female	106 (62.7%)	26 (28.3%)
	Male	63 (37.3%)	66 (71.7%)
Smoking Status	Current smoker	48 (28.4%)	30 (32.6%)
	Ex-smoker	80 (47.3%)	61 (66.3%)
	n/a	3 (1.8%)	
	Non-smoker	38 (22.5%)	1 (1.1%)

* 6th Edition UICC/AJCC classification criteria.

### Tiling path array comparative genomic hybridization

Array hybridization was performed as previously described [30], [87], [88]. Briefly, equal amounts (200–400 ng) of sample (extracted from either fresh-frozen or FFPE tissues) and single male reference genomic DNA were differentially labelled and hybridized to SMRT array v.2 (BCCRC Array Laboratory, Vancouver, BC), which is previously described to give optimal genome coverage [27], [89].

Hybridized arrays were imaged using a charge-coupled device (CCD) camera system and analyzed using *SoftWoRx Tracker Spot Analysis* software (Applied Precision, Issaquah, WA). Systematic biases were removed from all array data files using a stepwise normalization procedure as previously described [29], [32]. *SeeGH* software was used to combine replicates and visualize all data as log_2_ ratio plots [90], [91]. Stringently, all replicate spots with a standard deviation above 0.075 or signal to noise ratios below three were removed from further analysis. The probes were then positioned based on the human March 2006 (hg 18) genome assembly. Genomic imbalances (gains and losses) within each sample were identified using *aCGH-Smooth* [28] with lambda and breakpoint per chromosome settings at 6.75 and 100, respectively (as previously described) [30]. The resulting frequency of alteration was then determined for each lung cancer cell type as described previously [30].

### DNA Methylation Analysis

For 30 AC samples, 30 patient-matched non-malignant lung samples, 13 SqCC samples and 18 non-patient matched bronchial epithelia samples (all fresh-frozen samples), DNA methylation profiling was performed using the Illumina HumanMethylation27 chip. Five hundred nanograms of DNA from each sample were analyzed by this technology. Normalized β-values were obtained and only those with a detection *p*-value of ≤0.05 were used. When comparing tumor samples (AC/SqCC) and normal non-malignant samples (AC non-malignant parenchyma and bronchial epithelia), probes were deemed aberrantly methylated if the absolute difference between tumor and the average of the appropriate normal samples was ≥0.15.

### Comparison of subtype alteration frequencies

Regions of differential copy number alteration between AC and SqCC genomes were identified as follows. Each array element was scored as 1 (gain/amplification), 0 (neutral/retention), or −1 (loss/deleted) for each individual sample. Values for elements filtered based on quality control criteria were inferred by using neighbouring probes within 10 Mb. Probes were then aggregated into genomic regions if the similarity in copy number status between adjacent probes was at least 90% across all samples from the same subtype. The occurrence of copy number gain/amplification, loss/deletion, and retention at each locus was then compared between AC and SqCC data sets using the Fisher's exact test. Testing was performed using the *R* statistical computing environment on a 3×2 contingency table as previously described, generating a p-value for each probe [30]. A Benjamini-Hochberg multiple hypothesis testing correction based on the number of distinct regions was applied and resulting p-values ≤0.01 were considered significant. Adjacent regions within 1 Mb which matched both the direction of copy number difference and statistical significance were then merged. Finally, regions had to be altered in >20% of samples in a group and the difference between groups >10% to be considered.

A similar approach was used for determining subtype-specific DNA methylation alterations. Frequencies of hypermethylation and hypomethylation for each probe were compared using a Fisher's exact test, followed by a Benjamini-Hochberg multiple testing correction. A corrected *p*-value cut-off of *p*<0.05 was used to deem a probe differentially methylated between the two groups.

### Gene expression microarray analysis

Fresh-frozen lung tumors were obtained from Vancouver General Hospital as described above. Microdissection of tumor cells was performed and total RNA was isolated using RNeasy Mini Kits (Qiagen Inc., Mississauga, ON). Samples were labeled and hybridized to a custom Affymetrix microarray according to the manufacture's protocols (Affymetrix Inc., Santa Clara, CA). In addition, RNA was obtained from exfoliated bronchial cells of lung cancer free individuals obtained during fluorescence bronchoscopy [71]. All individuals were either current or former smokers. Expression profiles were generated for all cases using the Affymetrix U133 Plus 2 platform (Affymetrix Inc., Santa Clara, CA). All data was normalized using the Robust Multichip Average (RMA) algorithm in [92]. In addition, publically available datasets downloaded from the *Gene Expression Omnibus* were used: Affymetrix U133 Plus 2 expression data was downloaded for accession numbers GSE3141 [93] and GSE8894 [48].

### Statistical analysis of gene expression data

Gene expression probes were mapped to March 2006 (hg 18) genomic coordinates and those within the regions of copy number difference between the subtypes were determined. Comparisons between expression levels for AC and SqCC tumors were performed using the Mann-Whitney U test and computed with the *ranksum* function in *Matlab*. As the direction of gene expression difference was predicted to match the direction of copy number difference, one tailed *p*-values were calculated. A Benjamini-Hochberg multiple hypothesis testing correction was applied based on the total number of gene expression probes analyzed for each region. Probes with a corrected *p*-value≤0.001 were considered significant. If multiple probes mapped to the same gene, the one with the lowest p-value was used. Resulting genes were then mapped to the corresponding probes on the Affymetrix U133 Plus 2 array in order to compare their expression in a second set of NSCLC tumors (GSE3141 above) against normal bronchial epithelial cells. If multiple probes were present for a gene, the one with the strongest *p*-value was used. All comparisons were performed using a one-tailed t-test with unequal variances in *Excel* and genes with a p<0.001 were considered significant. The fold-change for tumors versus normal tissues was then determined in order to determine genes expressed in the direction predicted by copy number.

Principle component analysis was performed using expression data for the three independent tumor data sets (described above) in MATLAB. All genes of interest with probes on the corresponding arrays were used. Briefly, the first and second principal components were generated from the original dataset. In the subsequent validation in secondary datasets, these principal components are then used to weight the expression data for a gene based on the original distribution. The Receiver Operating Characteristic (ROC) area under the curve (AUC) analysis was performed to determine the ability of principle component 1 to separate the AC and SqCC samples into their appropriate histological groups. Calculations were performed using the *GraphPad Prism* software.

Connectivity Map (http://www.broad.mit.edu/cmap/) analysis was performed using the up and downregulated genes specific to each subtype as previously described [50].

### Survival analysis

Survival analysis was performed using the statistical toolbox in *Matlab*. Expression data for each gene were sorted and survival times were compared between the top 1/3 and bottom 1/3 in expression using a publicly available gene expression microarray dataset with survival data (Table S1). Two tailed *p*-values were generated using a Mantel-Cox log test and those <0.05 were considered significant. Kaplan-Meier plots were then generated for each gene of interest.

### Network identification

Functional identification of gene networks and canonical signalling pathways was performed using Ingenuity Pathway Analysis program (Ingenuity® Systems, www.ingenuity.com). AC and SqCC specific gene lists were imported as individual experiments using the Core Analysis tool. The analysis was performed using Ingenuity Knowledge Database with the Affymetrix U133 Plus 2 platform as the reference set and was limited to direct and indirect relationships.

### Human lung tissue tissue microarray case selection

To determine the expression of ERCC1, KEAP1 and SOX2 in primary NSCLC, we selected 330 NSCLCs (AC, n = 220; SqCC, n = 110) from surgically resected lung cancer specimens from the Lung Cancer Specialized Program of Research Excellence TissueBank at The University of Texas M.D. Anderson Cancer Center. We used archived, formalin-fixed, paraffin-embedded (FFPE) tumor tissue samples placed in tissue micro-array (TMA). The tumor tissue samples were collected between 1997 and 2003, and were histologically analyzed and classified using the 2004 WHO classification system [94]. The characteristics of these TMAs have been previously described in detail [95], [96].

### Immunohistochemical analysis

The immunohistochemical analysis was done using commercially available antibodies against KEAP1 (dilution1∶25; Proteintech, Chicago, IL), ERCC1 (dilution 1∶25; Labvision, Fremont, CA) and SOX2 (dilution 1∶50; R&D system, Minneapolis, MN). Immunohistochemical staining was done using an automated stainer (Dako, Inc.) with 5-µm-thick TMA sections from FFPE tissues. Tissue sections were deparaffinized and hydrated. Antigen retrieval was done in pH 6.0 citrate buffer in a decloaking chamber (121°C×30 seconds, 90°C×10 seconds) and washed on Tris buffer. Peroxide blocking was done at ambient temperature with 3% H2O2 in methanol. The slides were incubated with primary antibody (KEAP1 and ERCC1 for 60 minutes; SOX2 for 90 minutes) at ambient temperature and washed with Tris buffer, followed by incubation with biotin-labeled secondary antibody for 30 minutes (EnVision Dual Link System-HRP-Dako for KEAP1 and ERCC1; LSAB system-Dako for SOX2). The immunostaining was developed with 0.5% 3,3′- diaminobenzidine, freshly prepared with imidazole-HCl buffer (pH 7.5) containing hydrogen peroxide and an antimicrobial agent (Dako) for 5 minutes, and then the slides were counterstained with hematoxylin, dehydrated, and mounted.

Nuclear ERCC1, cytoplasmic KEAP1, and nuclear SOX2 expressions were quantified using a four-value intensity score (0, 1+, 2+, or 3+) and the percentage (0–100%) of the extent of reactivity. An immunohistochemical expression score was obtained by multiplying the intensity and reactivity extension values (range, 0–300), and these expression scores were used to determine expression levels.

### Trichostatin A dose-response analysis

The effect of HDAC inhibitor Trichostatin A, (Cayman Chemicals, Denver, CO, USA) on six NSCLC cell lines; three adenocarcinoma (H3255, H1395 and A549) and three SqCC lines (HCC95, HCC15 and H520) was assessed by cell viability assays. Cells were plated in triplicate in 96 well plates at optimal densities for growth (A549 at 2000 cells/well, HCC95, HCC15 and H520 at 3000 cells/well, and H3255 and H1395 at 6000 cells/well). Cells were subjected to a series of 2-fold dilutions of Trichostatin A prepared in cell growth media and DMSO. The experimental inhibitor concentrations ranged from 100 uM to 109 pM and the final DMSO concentration for treated and untreated (control) cells was 1%. Blank wells contained equal volumes of growth media with 1% DMSO. Cells were incubated for 72 hours at 37°C and then treated with 10 µl of Alamar Blue cell viability reagent (Invitrogen, Carlsbad, CA, USA) according to manufacturer's instructions. The reaction product was quantified by measuring absorbance at 570 nm with reference to 600 nm using an EMax plate reader (Molecular Devices, Sunnyvale, CA, USA). The response of treated cells was measured as a proportion of the viability of untreated cells, with the mean background subtracted treatment absorbance divided by the mean background subtracted untreated absorbance for each inhibitor concentration. Dose response curves and IC_50_ values were generated in Graph Pad v5 using the proportionate response of all 20 drug concentrations. Experiments were repeated in quadruplicate and differences in IC_50_ values were determined using a student's t-test with a p-value<0.05 considered significant.

## Supporting Information

Figure S1 **Principle component analysis of NSCLC cell lines using subtype-specific genes.**
(TIF)

Table S1 **Clinical Samples used in Analyses.**
(DOCX)

Table S2 **List of copy number alterations specific to each subtype.**
(XLSX)

Table S3 **SqCC-Specific Copy Number Alteration Present in CIS Cases.**
(XLSX)

Table S4 **Genes deregulated by copy number specifically in each subtype.**
(XLSX)

Table S5 **IPA network analysis of subtype specific copy number alteration target genes.**
(XLSX)

Table S6 **Genes deregulated by DNA methylation in each subtype.**
(XLSX)

Table S7 **IPA network analysis of subtype specific methylation target genes.**
(XLSX)

Table S8 **Genes disrupted by gene-dosage in one subtype and DNA methylation in the other.**
(XLSX)

Table S9 **Genes demonstrating concerted genetic, epigenetic and transcriptional disruption in SqCC.**
(XLSX)

Table S10 **Survival analysis of subtype-specific genes.**
(XLSX)

Table S11 **CMAP analysis of subtype-specific genes.**
(XLS)
