# Supplementary figures and images for: Divergent Genomic and Epigenomic Landscapes of Lung Cancer Subtypes Underscore the Selection of Different Oncogenic Pathways during Tumor Development
Source: PLoS One. 2012 May 21;7(5):e37775. doi: 10.1371/journal.pone.0037775 (PMC3357406; doi:10.1371/journal.pone.0037775)

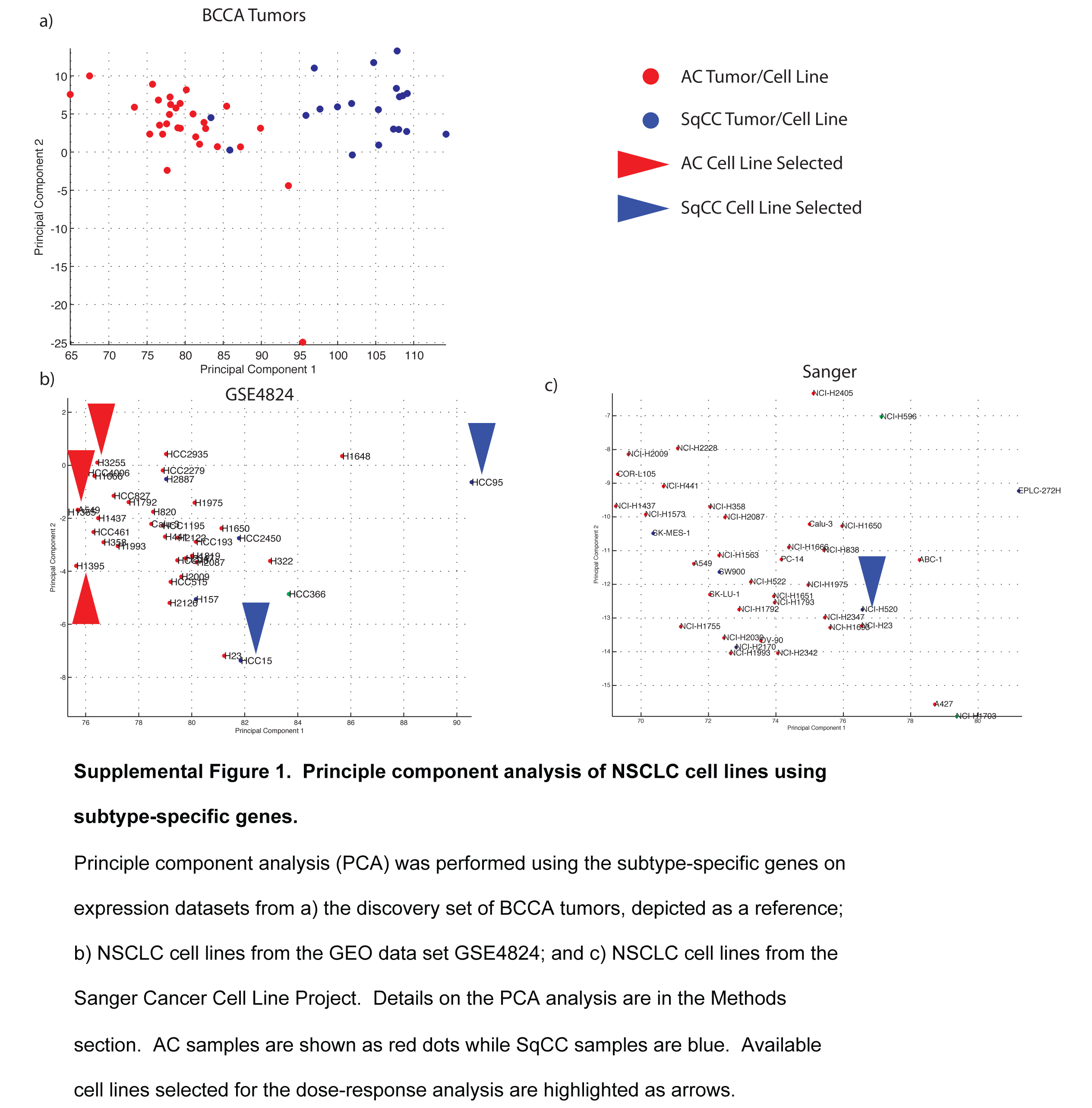

Supplement: Figure S1 — Principle component analysis of NSCLC cell lines using subtype-specific genes. (TIF) [file pone.0037775.s001.tif]
