# Supplementary material for: Divergent Genomic and Epigenomic Landscapes of Lung Cancer Subtypes Underscore the Selection of Different Oncogenic Pathways during Tumor Development
Source: PLoS One. 2012 May 21;7(5):e37775. doi: 10.1371/journal.pone.0037775 (PMC3357406; doi:10.1371/journal.pone.0037775)
Supplement: Table S1 — Clinical Samples used in Analyses. (DOCX) [file pone.0037775.s002.docx]

**Table S1:** Clinical Samples used in Analyses

| **Sample Set** | **Cohort** | **# of Samples** | **# AC** | **# SqCC** | **# Non-Neoplasitic Lung Tissue**^$^ | **Assay Type** |
| --- | --- | --- | --- | --- | --- | --- |
| 1 | BC Cancer Agency - Vancouver  St. Paul's Hospital - Vancouver  University Health Network - Toronto | 261 | 169 | 92 | 0 | Copy Number - BCCRC Whole Genome Tiling Path Array CGH |
| 2 | BC Cancer Agency - Vancouver | 49 | 29 | 20 | 0 | Gene Expression - Custom Affymetrix |
| 3 | GEO Duke University – GSE3141 | 111 | 58 | 53 | 0 | Gene Expression - Affymetrix GeneChip Human Genome U133 Plus 2.0 Array |
| 4 | BC Cancer Agency – Vancouver | 0 | 0 | 0 | 67 | Gene Expression - Affymetrix GeneChip Human Genome U133 Plus 2.0 Array |
| 5 | BC Cancer Agency – Vancouver | 92 | 30 | 13 | 48 | DNA Methylation - Illumina HumanMethylation27 chip |
| 6 | GEO Samsung Medical Center -GSE8894 | 138 | 62 | 76 | 0 | Gene Expression - Affymetrix GeneChip Human Genome U133 Plus 2.0 Array |

^$^ Non-neoplastic lung tissue includes exfoliated bronchial epithelial cells from cancer free individuals and non-malignant lung parenchyma
